# Effects of vitamin D-induced supernatant of placental explants from preeclamptic women on oxidative stress and nitric oxide bioavailability in human umbilical vein endothelial cells

**DOI:** 10.1590/1414-431X2020e11073

**Published:** 2021-05-24

**Authors:** P.R. Nunes, V.J. Gomes, V.C. Sandrim, J.C. Peraçoli, M.T.S. Peraçoli, M. Carlström

**Affiliations:** 1 Departamento de Ginecologia e Obstetrícia, Faculdade de Medicina de Botucatu, Universidade Estadual Paulista “Júlio de Mesquita Filho”, Botucatu, SP, Brasil; 2 Department of Physiology and Pharmacology, Karolinska Institutet, Stockholm, Sweden; 3 Departamento de Biofísica e Farmacologia, Instituto de Biociências, Universidade Estadual Paulista “Júlio de Mesquita Filho”, Botucatu, SP, Brasil

**Keywords:** Oxidative stress, Placental explants, Preeclampsia, Vitamin D

## Abstract

The study evaluated the effect of the supernatant of placental explants from preeclamptic (PE) and normotensive (NT) pregnant women after tissue treatment with or without vitamin D (VD) on oxidative stress and nitric oxide (NO) bioavailability in human umbilical vein endothelial cells (HUVEC). Placental explants were prepared from eight NT and eight PE women, and supernatants were obtained after incubation with or without hydrogen peroxide (H_2_O_2_) and/or VD. HUVEC were cultured for 24 h with supernatants, and the following parameters were analyzed in HUVEC cultures: NO, nitrate (NO_3_^-^), and nitrite (NO_2_^-^) levels, lipid peroxidation, and intracellular reactive oxygen species (ROS). Results showed that the production of NO_3_^-^, NO_2_^-^, malondialdehyde (MDA), and ROS were significantly higher in HUVEC treated with explant supernatant from PE compared to NT pregnant women, while the supernatant of PE explants treated with VD led to a decrease in these parameters. A significantly high production of NO was detected in HUVEC cultured with control supernatant of NT group, and in cultures treated with supernatant of PE explants treated with VD. Taken together, these results demonstrated that cultures of placental explants from PE women with VD treatment generated a supernatant that decreased oxidative stress and increased the bioavailability of NO in endothelial cells.

## Introduction

Preeclampsia (PE) is a specific human syndrome of pregnancy characterized as the main cause of morbidity, mortality, and preterm birth, affecting as many as 10% of all pregnancies. Clinical diagnosis is performed from the twentieth week of pregnancy or in the first days after delivery, based on the development of hypertension with or without proteinuria (1) in addition to maternal manifestations such as thrombocytopenia, impaired liver function, renal insufficiency, pulmonary edema, and new-onset cerebral or visual disturbances (2).

Nitric oxide (NO) is a key signaling molecule in the cardiovascular system, controlling vascular tone, neurotransmission, redox signaling, cellular respiration, and host defense (3). This molecule participates actively in the pregnancy processes such as trophoblast invasion and placental development, representing the main vasodilator in the placenta (4). Disturbances in the NO system, coupled with oxidative stress, contribute to vascular dysfunction in preeclamptic women (5). Oxidative stress, characterized by excessive formation of reactive oxygen species (ROS), can impair endothelial nitric oxide synthase (eNOS) function (6) and consequently decrease bioavailability of NO. Together with the excessive production of ROS, the placenta from preeclamptic women shows an intense inflammatory process. ROS are involved in the injuries signaling to the immune system (7) and can orchestrate the inflammatory response by the release of hydrogen peroxide (H_2_O_2_) from damaged tissues leading to the recruitment of leukocytes to the lesion site (8).

Vitamin D (VD) has several effects on the organism, modulating cardiovascular and immune cell functions. Concerning the innate and adaptive immune system, it can establish a more tolerogenic immune status, by its regulatory activities on the inflammatory response. During normal pregnancy, VD is produced by placental trophoblast cells and human decidua and is responsible for anti-inflammatory effects in various organs, including the placenta (9).

This hormone plays an important role in the implantation, placentation, and maintenance of healthy gestations. During human pregnancy, the conversion of inactive 25(OH)-D to the active form 1.25(OH)2-D is increased in the placenta demonstrating that this tissue and decidua are important in the bioactivation of VD (10). Recent studies have reported VD deficiency in pregnant women with PE (11,12) and others have shown an association between deficiency and the risk of developing PE, suggesting that supplementation may modulate the immune response in this pathology (13).

Recently, we demonstrated the *in vitro* activation of NLRP3 inflammasome in placental explants from normotensive (NT) pregnant women as a consequence of the H_2_O_2_-induced cellular stress, which is initiated by ROS release, as well as increased gene expression of inflammatory cytokines (14). Furthermore, the increase of ROS formation could decrease the bioavailability of NO, since some researchers have suggested that PE should be characterized by a disruption of vascular dilation mediated by NO and disturbed by ROS (15). Therefore, the use of immunomodulatory substances like VD for the *in vitro* treatment of explants may lead to a better understanding of the systemic inflammation in PE and possibly propose alternative ways to treat this syndrome.

Considering that the placenta of women with PE shows oxidative stress, exacerbated inflammation, and NO system imbalance, this study aimed to evaluate the effect of the supernatant of placental explants from PE and NT pregnant women treated with or without VD on oxidative stress and NO bioavailability in human umbilical vein endothelial cells (HUVEC). We intended to observe whether the employment of substances with an immunomodulatory effect such as VD on placental tissue can be used to reduce oxidative stress in endothelial cells.

## Material and Methods

The methodology employed in this study is demonstrated in a schematic figure (Supplementary Figure S1).

### Study population and ethics statement

Placentas and blood from 8 NT and 8 PE pregnant women were collected. These pregnant women were admitted to the Obstetrics Unit of Botucatu Medical School, Sao Paulo State University, Botucatu, SP, Brazil, between November 2017 and August 2018. Gestational age was calculated from the last menstrual period and confirmed by ultrasound dating. A pregnant woman was considered preeclamptic when, without a history of hypertension, she developed hypertension (≥140/90 mmHg) associated or not with proteinuria (≥300 mg in 24-h urine) after the 20th week of gestation (1). NT pregnant women did not present a personal history of hypertensive disorders before or during pregnancy. Proteinuria in 24-h urine was analyzed by a colorimetric method, the Technicon RA-XT automation system (Asinteg, Argentina), and uric acid was assessed by uric acid enzymatic Trinder (Biotrol Diagnostic, India) in the Clinical Laboratory of Botucatu Medical School.

Exclusion criteria included chronic hypertension, multiple gestations, prior preeclampsia, illicit drug use, and preexisting medical conditions such as diabetes, cancer, acute infectious disease, and cardiovascular, autoimmune, renal, and hepatic diseases. The study was approved by the Ethics Committee of the Botucatu Medical School, and written informed consent was obtained from all women involved in the study (Protocol number: 3.383.792). This work was carried out under the Code of Ethics of the World Medical Association (Declaration of Helsinki). Also, all mandatory laboratory health and safety procedures were complied with in the course of conducting any experimental work reported in this paper and all experiments were performed following relevant guidelines and regulations.

### Collection of placental tissue

Placentas were collected at delivery by elective cesarean section. Immediately after delivery, all placentas considered for the study were examined macroscopically and processed within 10 min. Fragments of approximately 5×5 cm were immediately removed from the central region of the placenta, constituting samples in contact with the maternal side (basal plate). After this initial collection, smaller fragments were washed in phosphate buffered saline (PBS) and separated from the decidual layer that is normally adhered to the basal plate. The terminal portions of the villi were observed in PBS (the villi were seen floating freely in the liquid) and were dissected into small sections to constitute explants.

### Culture of placental explants with hydrogen peroxide and vitamin D

Placental tissue was obtained according to a previous study (14). Briefly, a total of 11 mg of human villous tissue was cultured in RPMI 1640 culture medium (Thermo Fisher, USA) supplemented with 2 mM L-glutamine (Sigma-Aldrich, USA), 40 mg/mL antibiotic/antimycotic (Sigma-Aldrich), and 10% fetal bovine serum (Gibco BRL Life Technologies, The Netherlands) in 24-well plates (SPL Life Sciences, Korea) during 24 h for stabilization. After stabilization, placental explants from PE and NT pregnant women were cultured for 24 h with or without H_2_O_2_ (100 μM) (Sigma-Aldrich) as a stimulus for oxidative stress induction, and with or without VD (100 nM) (Sigma-Aldrich), considered an immunomodulator. Placental explant supernatants obtained were stored at -80°C for later experiments and analyses.

### Cytokine determinations

The concentrations of interleukin (IL)-1β, tumor necrosis factor (TNF)-α, and IL-18 in supernatants of placental explants from NT and PE women, obtained after treatment with or without H_2_O_2_ and VD, were determined by Quantikine ELISA kits (R&D Systems, USA) according to the manufacturer's instructions. Assay sensitivity limits were 1.0 pg/mL for IL-1β, 1.6 pg/mL for TNF-α, and 5.15 pg/mL for IL-18.

### Vitamin D and vitamin D receptor (VDR) determination

*Blood sampling and vitamin D determination*. Peripheral blood (10 mL) was obtained by venipuncture from the antecubital vein of 8 PE women at the time of PE diagnosis and of 8 NT pregnant women at the time they were matched to gestational age with PE women. Blood was collected into plastic tubes containing 5% EDTA. After blood centrifugation at 4°C for 10 min at 1,200 *g*, the plasma fraction was removed and aliquots were stored at -80°C until vitamin D determination.

Vitamin D, 25(OH)D, was determined by the automated chemiluminescence microparticle immunoassay (CMIA) with an Architect 25-OH Vitamin D assay kit, by the Architect^®^ i2000 analyzer (Abbott^®^, USA ). The analytical sensitivity was 1.9 ng/mL and the coefficient of variation within and between assays was <10%, as described in the kit. The reference range was 0.0-160.0 ng/mL, according to the method. Values ≥30 ng/mL were considered sufficient, from 21 to 29 ng/mL insufficient, and <20 ng/mL deficient (16).

### VDR gene expression

Placental explants from 8 PE and 8 NT pregnant women were employed to determine the expression of VDR at the transcriptional level. Total RNA was extracted from the placental explants after culture using the Total RNA Purification Kit (Norgen Biotek Corp., Canada) following the manufacturer's protocol, and the reverse transcription-coupled polymerase chain reaction (qPCR) was performed as described previously (14). Briefly, isolated RNA was treated with DNAse I Amp Grade (Invitrogen, USA). Subsequently, the synthesis of complementary DNA (cDNA) was conducted using ImProm-II TM Reverse Transcription System, according to the manufacturer's protocol (Promega, USA). The qPCR was performed using RT GoTaq-qPCR Master Mix (Promega, USA) and the variants of the studied targets were aligned in the MEGA 5.1 program (http://www.megasoftware.net) and each primer was subsequently selected by the software Primer-BLAST (NIH, USA). Primers located in exon-exon junctions guarantee the purity of the reaction, namely the absence of any genomic DNA that may contaminate it. The VDR primer sequence used in this study was (590)TGGAGACTTTGACCGGAACG(609) (704)GCTTCGCCTGAAGAAGCCT(686). Each reaction was set in duplicate and the conditions for the qPCR were as follows: initial denaturation at 96°C for 2 min and then 40 cycles at 95°C for 15 s and 60°C for 60 s, followed by a melting curve. Expression values of the analyzed transcripts were normalized to that of the enzyme-encoding glyceraldehyde-3-phosphate dehydrogenase gene (GAPDH) as follows: (684)CGTGGAAGGACTCATGACCA(703) (801)GGCAGGGATGATGTTCTGGA(782). The calculation of the differential expression of selected genes was carried out by the data processing method compared with a standard curve (17). To analyze the relative expression, after the analysis of gene expression, we chose an RNA sample obtained from each group, which received a relative value of 100. All other samples received values for that sample.

### HUVEC culture and incubation with supernatant of placental explants

HUVEC (Lonza, Switzerland) were acquired with a certification proving that the cells were from the designated type. All experiments were performed using cells in the sixth passage and in quintuplicates. HUVEC were cultured until reaching 80% confluence and then incubated for 24 h in medium 199 (Gibco BRL Life Technologies) with 20% (v/v) supernatant pool from placental explants of PE women, and from NT explants previously cultured with or without H_2_O_2_ and/or VD as described above. The cells and HUVEC media were used to perform the assays. PrestoBlue^®^ Cell Viability Reagent (Invitrogen) was employed to determine whether the supernatants pre-treated with H_2_O_2_ and/or VD were harmful to HUVEC showing cell viability.

### Measurement of nitrate, nitrite, and NO species

Nitrate and nitrite levels produced by HUVEC after placental explant supernatant incubation for 24 h were assessed in HUVEC supernatant using a high-performance liquid chromatography (HPLC) system (ENO-20; Eicom, USA) as previously described (18). The ENO-20's high sensitivity and specificity were accomplished with the combination of a diazo coupling method and chromatography. The level of the diazo compound was measured by absorbance at 540 nm using a Spectramax iD3 multi-mode microplate reader (Hidex, Lablogic Systems, UK).

A total of 5×10^4^ HUVEC per well were plated onto a black 96-well plate (Sigma-Aldrich). After 24 h of incubation with placental explant supernatants, the cells were washed with 95 µL of PBS and incubated for 30 min at 37°C. After this period, cells were loaded with 5 µL of DAF-FM™ (10 µM) (Sigma-Aldrich) and read for 60 min in 37°C using Spectramax iD3. The fluorescence signal was measured in a microplate reader (excitation 495 nm, emission 535 nm) and is reported as arbitrary units.

### Measurement of lipid peroxidation - TBARS

Levels of lipid peroxidation in the supernatant of HUVEC cultured with pre-treated placental explants supernatant for 24 h were measured by thiobarbituric acid reactive substances (TBARS). An aliquot of 100 μL of supernatant was mixed with 200 μL of 10% cold trichloroacetic acid (TCA, Sigma-Aldrich), before being placed on ice for 15 min to precipitate protein. During this time, standards were prepared in a serial dilution with 1,1,1,3-tetramethoxypropane (TMP, C_7_H_16_O_4_, 133.75 mM, Sigma-Aldrich). Then, the samples with TCA were centrifuged at 2,200 *g* for 15 min at 4°C. After centrifugation, 200 μL of the supernatants and standards were placed in cryotubes with an equal volume of 0.67% thiobarbituric acid (TBA, Sigma-Aldrich) and boiled at 95°C for 50 min. Finally, the tubes were placed on ice for 3 min. Standards and samples were placed on a 96-well plate and the absorbance was measured at 532 nm (Spectramax iD3) and the TBARS values were calculated using the malondialdehyde (MDA) standard curve. The values are reported as nanomoles of MDA per mL.

### Levels of intracellular ROS

Intracellular ROS was quantified in the supernatant of HUVEC cultured with pre-treated placental explants supernatant using 2′-7′-dichlorodihydrofluorescein diacetate (DCFH-DA; Sigma-Aldrich) by fluorescence with 2V,7V-dichlorofluorescein diacetate. Tert-butyl hydroperoxide (tBHP) at 1000 µM was added 2 h before reading as a positive control. After 24 h of treatment with placental explant supernatants, HUVEC were incubated with 100 µM DCFH-DA diluted in dimethyl sulfoxide (DMSO) for 30 min at 37°C. Then, cells were washed with PBS (pH 7.4) and the relative levels of fluorescence were quantified in a spectrophotofluorimeter (Hitachi F-4500, Japan, 485 nm excitation and 520 nm emission). The measured fluorescence values are reported as fluorescence intensity.

### Statistical analysis

Comparisons between groups were assessed by nonparametric tests (Mann-Whitney U test) and parametric analysis of variance (ANOVA) followed by Bonferroni's multiple comparison test. Results were evaluated using the statistical program PRISM (Graph Prism, version 6.01, GraphPad, USA) and statistical significance was accepted at P<0.05.

## Results

### Clinical and laboratory characteristics showed worse clinical outcomes in PE women

The differences between clinical and laboratory data of PE and NT pregnant women are reported in Table 1. There was no statistical difference in age between the groups. However, gestational age was lower in women with PE compared to NT. As expected, PE had worse clinical outcomes compared to NT pregnant women, and were associated with hypertension, hyperuricemia, and proteinuria.

**Table 1:** Characteristics of the pregnant women.

Variable	Normotensive (n=8)	Preeclampsia (n=8)
Age (years)	29 (18-41)	28 (16-39)
Gestational age (weeks)	40 (36-41)	34 (30-39)*
Systolic blood pressure (mmHg)	120 (110-120)	155 (140-70)*
Diastolic blood pressure (mmHg)	70 (60-80)	100 (90-110)*
Uric acid (mg/dL)	3.2 (2.3-4.7)	5.7 (3.7-6.9)*
Proteinuria (mg/24 h)	<300	730 (300-7540)*

Data are reported as median (minimum-maximum). *P<0.05 *vs* normotensive (Mann-Whitney U test).

### VD and VDR were lower in plasma and placental explants from PE women

**Figure 1:**
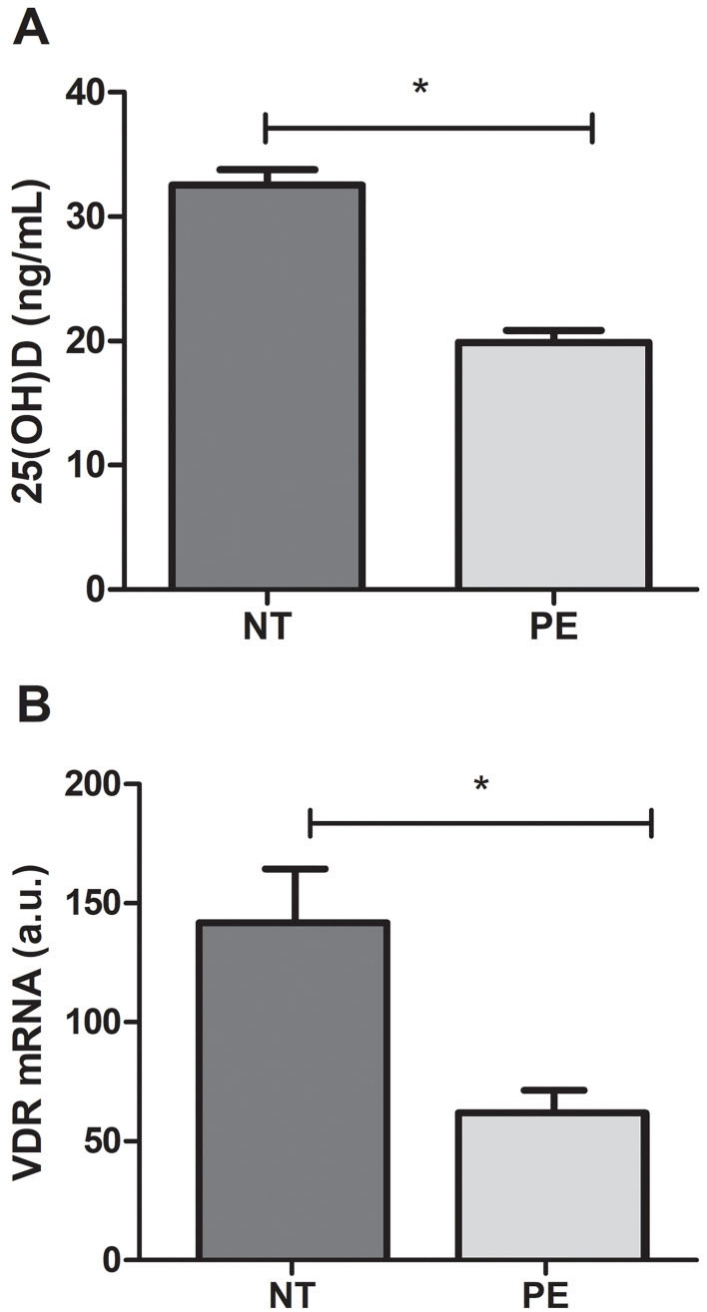
Levels of vitamin 25 (OH) D in plasma (**A**) and vitamin D receptor (VDR) gene expression (**B**) in the placental explants from preeclamptic (PE) (n=8) and normotensive (NT) pregnant women (n=8). Data are reported as means±SD. *P<0.05 (Mann-Whitney U test). a.u.: arbitrary units.

Figure 1A shows vitamin D 25 (OH)D levels in the plasma of PE and NT pregnant women. Figure 1B presents VDR gene expression in PE and NT placental explants. Both the VD levels and the receptor's gene expression were significantly lower in pregnant women with PE.

### Inflammatory cytokines were lower in supernatant from explants treated with VD

**Figure 2:**
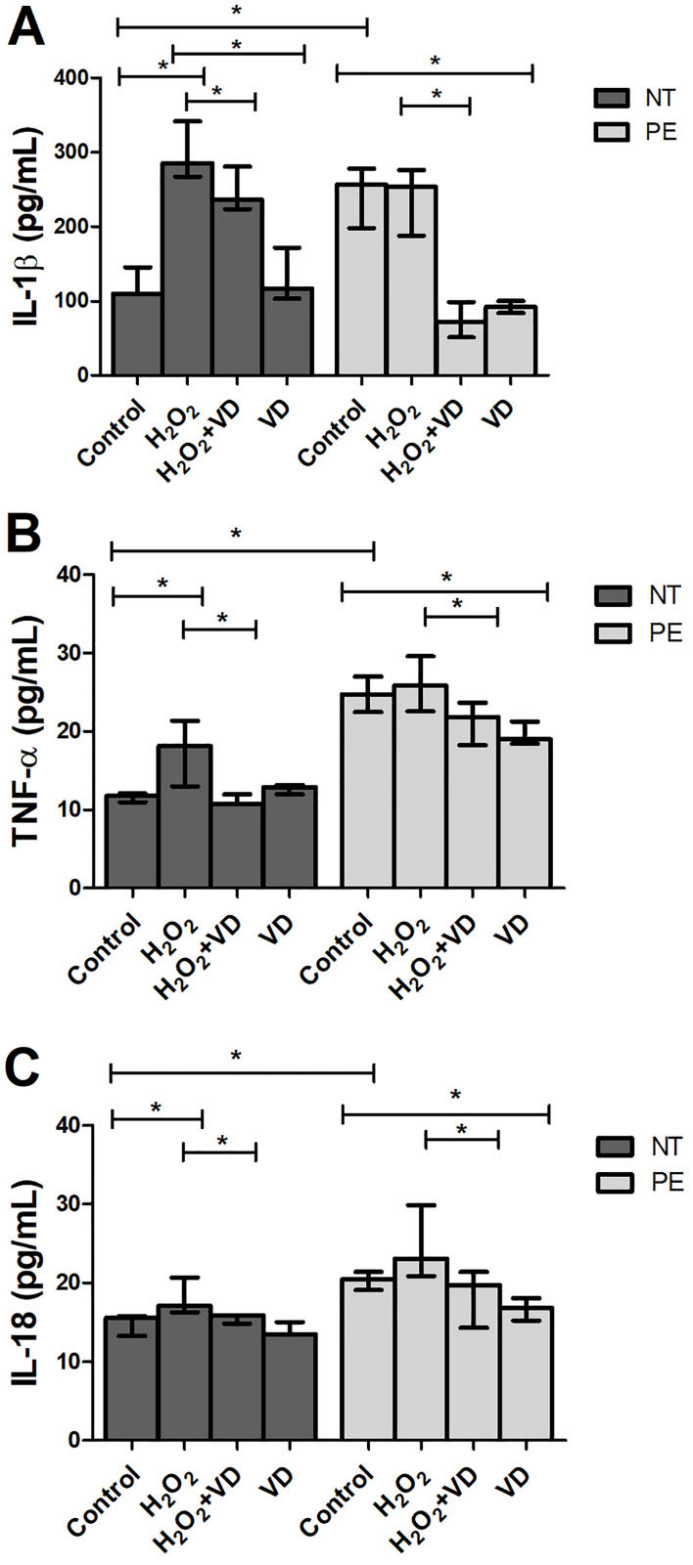
Concentration of cytokines in supernatant of placental explants from preeclamptic (PE) (n=8) and normotensive (NT) pregnant women (n=8) under different treatments. Data are reported as median with range. *P<0.05 (Mann-Whitney U test). IL-1β: interleukin-1 beta; TNF-α: tumor necrosis factor-alpha; IL-18: interleukin-18; VD: vitamin D.

Figure 2 shows the concentrations of cytokines in supernatant of placental explants from PE and NT pregnant women cultured in the absence or presence of H_2_O_2_ and VD. Protein expression of IL-1β, TNF-α, and IL-18 was significantly higher in supernatants from control cultures of PE compared to NT pregnant women. In the PE group, treatment with VD showed decreased expression of IL-1β, TNF-α, and IL-18 compared to control explants supernatant. The H_2_O_2_+VD treatment showed lower protein expression of IL-1β, TNF-α, and IL-18 compared to H_2_O_2_ treatment in cultures of NT and PE explants.

### Cell viability of HUVEC when treated with supernatants

After performing the dosages of VD and cytokines, the supernatant of PE and NT explants was used to treat the cultures of HUVEC. Before carrying out the culture, a viability test was performed to observe if the supernatants were harmful to the cells. HUVEC viability after 24-h culture with placental explant supernatant from PE and NT women pre-treated with H_2_O_2_, H_2_O_2_+VD, and VD is presented in Supplementary Figure S2. No significant differences were found between treatments regarding cell viability.

### Levels of nitrite and nitrate were significantly lower in HUVEC treated with supernatant of PE explants cultured with VD

**Figure 3:**
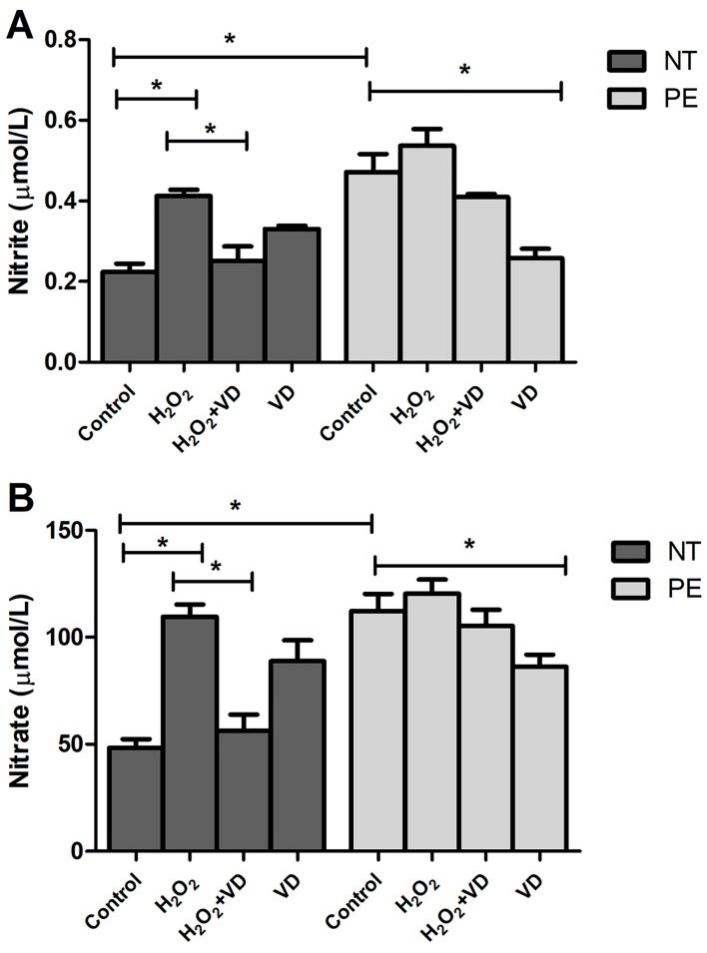
Levels of nitrite (**A**) and nitrate (**B**) in the supernatant of human umbilical vein endothelial cells treated with supernatant of placental explants from preeclamptic (PE) (n=8) and normotensive (NT) pregnant women (n=8), cultured in the absence (Control) or presence of H_2_O_2_, H_2_O_2_+VD, and VD. Data are reported as means±SD for one independent experiment, performed in quintuplicates. *P<0.05 (ANOVA followed by Bonferroni's multiple comparison test). VD: vitamin D.

Figure 3 shows nitrite and nitrate concentrations in supernatant of HUVEC after culture with supernatants of PE and NT placental explants pre-treated with H_2_O_2_, H_2_O_2+_VD, and VD. Levels of nitrite and nitrate were significantly higher in HUVEC cultures treated with PE control supernatant compared to NT control. Similarly, we observed higher levels of nitrite and nitrate in HUVEC cultures submitted to treatment with supernatant of placental explants from NT women treated with H_2_O_2_ compared to control supernatant. On the other hand, treatment of HUVEC with supernatant of PE explants cultured with VD led to a decrease in nitrite and nitrate levels compared to control supernatant, and also in the treatment with supernatant of placental explant of NT women cultured with H_2_O_2_+VD compared to only H_2_O_2_.

### HUVEC treated with NT supernatant produced higher levels of NO

**Figure 4:**
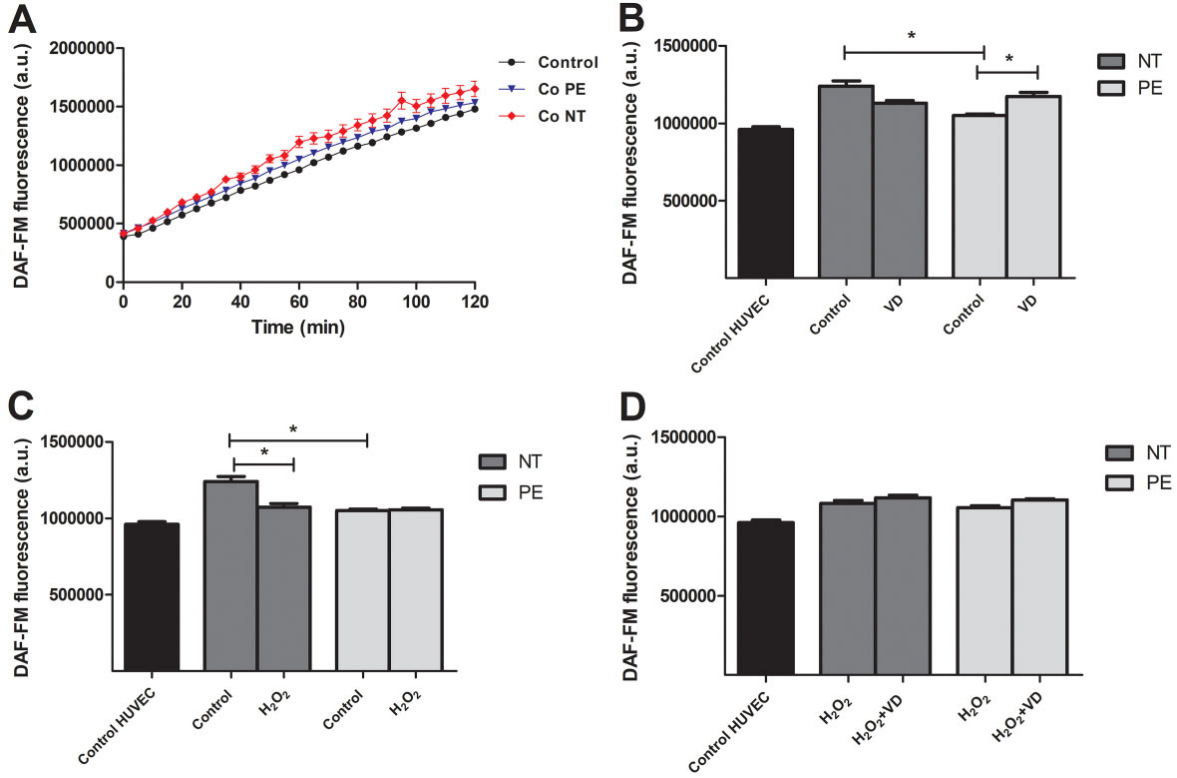
Nitric oxide fluorescence signal measured using DAF-FM™ after human umbilical vein endothelial cells (HUVEC) were exposed to placental explant supernatants from preeclamptic (PE) (n=8) and normotensive (NT) pregnant women (n=8). **A**, Control, Control NT, and Control PE; **B**, effect of treatment with vitamin D (VD) at 60 min; **C**, effect of treatment with H_2_O_2_ at 60 min; and **D**, effect of treatment with H_2_O_2_+VD at 60 min. Data are reported as means±SD for one independent experiment, performed in quintuplicates. *P<0.05 (ANOVA followed by Bonferroni's multiple comparison test). a.u.: arbitrary units.

Figure 4A shows the NO fluorescence signal at 120 min after HUVEC were exposed to placental explant supernatants (Control NT and Control PE). There was a significant difference between HUVEC treated with Control NT supernatant compared to cells exposed to Control PE supernatant, with higher NO production in Control NT supernatant-treated cells. Figure 4B-D shows NO generation by HUVEC after 60 min of incubation with DAF-FM™ after treatment with supernatant of placental explants. During this time of incubation (60 min), a significantly higher NO production by HUVEC treated with Control NT supernatant compared to Control PE supernatant-treated cells as well as a high production of NO in VD PE supernatant treated cells compared to Control PE were observed (Figure 4B). When cultured with pre-treated H_2_O_2_ NT supernatant, the cells showed lower NO production (Figure 4C). There was no significant difference between the production of NO in H_2_O_2_ supernatant treated cells compared to H_2_O_2_ +VD (Figure 4D).

### Measurement of lipid peroxidation - TBARS

**Figure 5:**
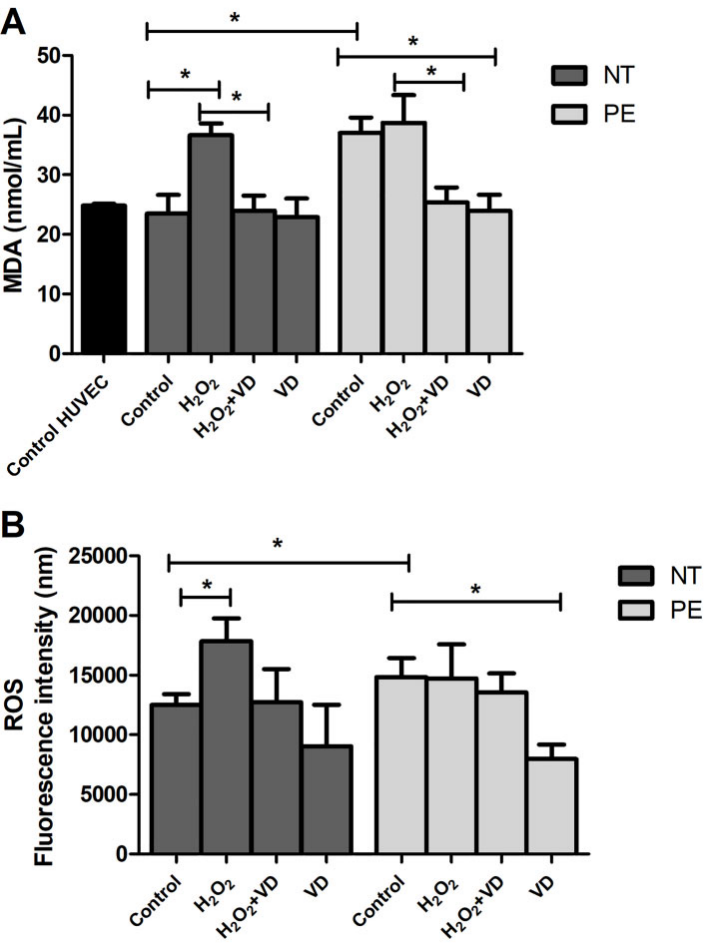
Malondialdehyde (MDA) levels (**A**) and levels of intracellular reactive oxygen species (ROS) (**B**) evaluated by fluorescence of 2,7-dichlorodihydrofluorescein diacetate (DCFH-DA) in the supernatant of human umbilical vein endothelial cells (HUVEC) treated with supernatant of placental explants from preeclamptic (PE) (n=8) and normotensive (NT) pregnant women (n=8), cultured in the absence (Control) or presence of H_2_O_2_, H_2_O_2_+vitamin D (VD), and VD. Data are reported as means±SD. *P<0.05 (ANOVA followed by Bonferroni's multiple comparison test).

The levels of MDA are shown in Figure 5A. Significantly higher MDA production was detected in HUVEC treated with Control PE explant supernatants compared to Control NT as well as compared to the VD PE group. MDA levels were significantly higher in the generated supernatant of HUVEC treated with H_2_O_2_ NT supernatant compared to Control NT and H_2_O_2_+VD NT, as well as in the H_2_O_2_ PE-treated group compared to H_2_O_2_+VD PE.

### Supernatants from PE explants cultured with vitamin D decreased levels of intracellular ROS in HUVEC

Figure 5B shows the ROS levels in the groups. Oxidative stress was higher in HUVEC cultured with PE control supernatant compared to the NT one. Supernatants from NT placental explants cultured with H_2_O_2_ increased oxidative stress in HUVEC, while supernatants from PE explants cultured with vitamin D decreased oxidative stress in these cells.

## Discussion

In the present study, we demonstrated that placental explants from PE women cultured without stimulus produced endogenous levels of the proinflammatory cytokines IL-1β, TNF-α, and IL-18. These results are in accordance with our previous studies showing a significant increase in NLRP3 inflammasome, caspase-1, IL-1β, and TNF-α in placental villi and in placental homogenate from PE women compared to NT pregnant women (19) and suggested the involvement of the placenta in the exaggerated inflammatory state that characterizes PE. In addition, this inflammatory process and the intense oxidative stress originated in the placenta may give rise to endothelial dysfunction in PE (7). Therefore, we evaluated the effect of the supernatant from placental explants of PE and NT pregnant women treated with or without VD on oxidative stress and NO bioavailability in HUVEC. In our understanding, no studies have evaluated the effect of these supernatants on endothelial cells function. Thus, we studied placentas from NT pregnant women at term and placentas of PE women in the last trimester of pregnancy, at the time of delivery.

The present study demonstrated that HUVEC cultured with endogenous supernatants of non-treated placental explants from PE women produced increased levels of NO_2_^-^, NO_3_^-^, MDA, and ROS. These results suggest that the endogenous activation state of PE placental tissues triggered oxidative stress in these endothelial cells. In opposition, significantly higher production of NO was detected after HUVEC treatment with control supernatant of NT placental explants compared with the PE group, as well as after treatment with supernatant obtained from PE explants cultured with VD. Thus, these results demonstrated that HUVEC treated with supernatant from placental explants pre-cultured with VD showed a decrease in oxidative stress and an increase in the bioavailability of NO. This hypothesis may be raised since the concentration of IL-1β, TNF-α, and IL-18 in the supernatant of placental explants from preeclamptic women decreased after treatment with VD, showing its modulatory effect on inflammatory cytokines production, particularly for its regulatory activities on the inflammatory response (20).

It has been proposed that the decrease in NO production in the placenta could cause abnormal tissue perfusion, which is observed in PE states. Regarding systemic production of NO, it was shown that the vasodilation of the brachial artery mediated by NO-dependent flow was approximately three times lower in pregnant women with PE compared to normal pregnant women (21). The NO system is also deranged in PE, as NO is a potent vasodilator, acting in order to induce relaxation in vascular smooth muscle cells (22). Decreased levels of NO have been reported in PE (23), and could be correlated with metabolic changes, such as hypertension, proteinuria, and platelet dysfunction (24).

Oxidative stress is an important phenomenon in PE (25) that could result from injury caused by hypoxia and reperfusion (26) and/or deficiency of antioxidant defenses (27). Peraçoli et al. (28) reported the endogenous activation of monocytes of pregnant women with PE, demonstrated by correlation among high production of superoxide anion (O_2_^-^), hydrogen peroxide (H_2_O_2_), and TNF-α by these cells, and high serum levels of uric acid, contributing to the enhanced oxidative and inflammatory state characteristic of PE. Hyperuricemia was also detected in the preeclamptic women in the present study, associated with high production of inflammatory cytokines by placental explants. According to other authors, production of uric acid concomitant with O_2_- generation decreases NO bioavailability, leading to endothelial dysfunction (29 [Bibr B30]). On the other hand, uric acid is also known as a powerful antioxidant through scavenging radical species and could also be regarded as a compensatory mechanism to counteract oxidative stress (29–31). Therefore, as hyperuricemia is a frequent finding in severe cases of PE, studies on oxidant and antioxidant properties of uric acid are of utmost importance to understand the pathophysiology of PE.

Several studies have been trying to show the effects of antioxidant therapy in women with PE. A systematic review performed by Rumbold et al. (32) concluded that supplementation with any antioxidant during pregnancy compared with control or placebo is associated with a reduced risk of developing PE. Similarly, Vadillo-Ortega et al. (33) showed that antioxidants in combination with L-arginine are effective in cases of PE risk. Evidence of association between vitamin D status and PE has been shown in various studies, but clinical trials did not show an independent effect of supplementation in cases of PE prevention. However, problems regarding dose, timing, and duration of supplementation with VD have not been completely explored (34).

Some authors have already shown that normal pregnancy is associated with an increase in oxidative stress and lipid peroxidation, but antioxidants also increase (35). In opposition, women with PE present an insufficient production of antioxidants to offset the increase in oxidative stress and lipid peroxidation (36).

An important oxidative stress biomarker, MDA is a product of fatty acid oxidation and an indicator of lipid peroxidation. Some authors showed increased MDA levels in the blood of pregnant women with PE (37). MDA binds to TBARS, which are also elevated in the blood of women with PE (37), reflecting an oxidative stress status. In the present study, we observed a significant increase in MDA and intracellular ROS in HUVEC treated with supernatant control from placental explants of PE compared to NT. On the other hand, the treatment with VD supernatant decreased these levels, demonstrating the antioxidant and anti-inflammatory effect. Wimalawansa (38) highlighted the effects of VD as one of the key controllers of systemic inflammation and oxidative stress, downregulating oxidative stress, cell and tissue damage, and the aging process. In the same way, the hypovitaminosis is enhances oxidative stress and systemic inflammation. VD is also known as a potent anti-oxidant that facilitates balanced mitochondrial activities, preventing oxidative stress-related protein oxidation, lipid peroxidation, and DNA damage (38). Our findings are consistent with previous studies suggesting that the overproduction of free radicals and lipid peroxidation are important factors in the pathogenesis of preeclampsia (39). In summary, oxidative stress could contribute to the cytotoxic mechanisms in PE, inducing cellular damage, leading to endothelial cell injury, inflammation, and angiogenic imbalance (40).

In conclusion, our results suggest that placental explants of preeclamptic women treated with VD can decrease oxidative stress and lipid peroxidation, as well as increase the bioavailability of NO in HUVEC.

## Supplementary Material

Click here to view [pdf].
